# Exploring within and between associations of momentary mindfulness and emotion regulation and the moderating effects of mental health among adolescents

**DOI:** 10.1111/jora.70114

**Published:** 2026-01-01

**Authors:** Reagan L. Miller‐Chagnon, Mark A. Prince, Shelley A. Haddock, Toni S. Zimmerman

**Affiliations:** ^1^ Department of Psychology, College of Natural Sciences Colorado State University Fort Collins Colorado USA; ^2^ Keck School of Medicine, Department of Psychiatry and the Behavioral Sciences University of Southern California Los Angeles California USA; ^3^ Department of Human Development & Family Studies, College of Health & Human Sciences Colorado State University Fort Collins Colorado USA

**Keywords:** adolescence, EMA, emotion regulation, mental health, mindfulness

## Abstract

Greater mindfulness is thought to give rise to more positive psychological health through more adaptive emotion regulation. While there is extensive evidence linking higher average mindfulness to lower average emotion regulation difficulties, less is known about the momentary‐level patterns that occur within individuals. Additionally, it is unclear if the relationship between mindfulness and emotion regulation changes as a function of adolescents' mental health status. This study sought to fill these gaps by investigating the between‐ and within‐person concurrent (same moment) and prospective (next moment) effects of mindfulness on emotion regulation among adolescents exposed to chronic stressors. This study also explored the moderating effects of mental health symptoms. Seventy‐nine participants who were 10–18 years old (*M* = 13.81; *SD* = 2.16; 56% male; 62% non‐Hispanic White) completed ecological momentary assessments (EMA) three times a day for 7 days. Dynamic structural equation modeling revealed that between‐ and within‐person mindfulness was inversely associated with concurrent, but not prospective, emotion regulation difficulties. Post‐traumatic stress disorder (PTSD) and internalizing symptoms also moderated concurrent and prospective within‐person associations. Individuals with higher PTSD and internalizing symptoms experienced lower emotion regulation difficulties during moments of greater‐than‐average mindfulness. Adolescents with higher PTSD symptoms also experienced fewer difficulties regulating their emotions in moments that followed greater mindful nonjudgment. These results highlight that greater momentary mindfulness may be beneficial for emotion regulation within the same moment and across the day for adolescents with mental health difficulties which has meaningful implications for just‐in‐time interventions. Future research may benefit from incorporating additional EMA measurements to explore the finer grained, short‐term dynamics between mindfulness and emotion regulation.

## INTRODUCTION

Greater mindfulness, a trait and state‐like characteristic of paying attention on purpose to the present moment with nonjudgment (Kabat‐Zinn, 1994), is consistently associated with fewer internalizing and externalizing symptoms among adolescents (e.g., Cortazar & Calvete, 2019; Greco et al., 2011; Pepping et al., 2016; Royuela‐Colomer et al., 2021), which are two broad classifications of behavioral, emotional, and social problems (Achenbach et al., 2016). Internalizing problems are characterized by symptoms of depression and anxiety, while externalizing problems represent impulsive behaviors and disruptive conduct (Achenbach et al., 2016). Similarly, adolescents with higher mindfulness also tend to experience fewer post‐traumatic stress disorder (PTSD) symptoms (Liu et al., 2022; Schmitz et al., 2021) and attention problems (Siebelink et al., 2019). One proposed pathway through which mindfulness is thought to give rise to more positive psychological health is through the promotion of adaptive emotion regulation (Guendelman et al., 2017; Roemer et al., 2015). Emotion regulation is defined as the process of effectively managing and expressing one's emotions and it is central to maintaining more positive mental health (Gross, 2014). The capacity for emotion regulation increases across adolescence (Steinberg, 2014) and adolescents who experience difficulties adaptively regulating their emotions often experience more persistent distress in the form of anxiety and depression symptoms (Aldao et al., 2010; Sheppes et al., 2015) as well as greater aggression (Röll et al., 2012). Research suggests that adolescents with higher trait mindfulness also experience fewer emotion regulation difficulties compared with those with lower trait mindfulness (Ma & Fang, 2019; Pepping et al., 2016). Despite evidence that mindfulness and emotion regulation are dynamic processes that unfold over time in response to emotion‐eliciting events (Bai et al., 2020; Miller et al., 2024), less is known about momentary‐level patterns that occur at the internal individual level. Further, both mindfulness and emotion regulation are inversely associated with adolescent psychopathology (Cortazar & Calvete, 2019; Pepping et al., 2016; Sheppes et al., 2015), but the moderating effect of mental health problems on the relationship between mindfulness and emotion regulation has yet to be explored. By clarifying if moments of greater mindfulness may give rise to fewer emotion regulation difficulties and by exploring if these associations change as a function of an individual's mental health, we may be more capable of accurately characterizing the utility of mindfulness for emotion regulation among adolescents with mental health difficulties. We may also gain knowledge about how adolescents respond to their lived environment. To fill these gaps in the literature, the current study sought to investigate the between‐ and within‐person concurrent (same moment) and prospective (next moment) effects of mindfulness on emotion regulation difficulties among adolescents exposed to chronic stressors. This study also explored the moderating effects of internalizing and externalizing symptoms, PTSD symptoms and attention problems on concurrent and prospective associations.

## THEORETICAL UNDERPINNINGS BETWEEN MINDFULNESS AND EMOTION REGULATION

A robust body of theoretical and empirical literature supports the conceptual link between mindfulness and emotion regulation. The monitor and acceptance (MAT) theory posits that the two core components of mindfulness, attention and nonjudgment, underlie and may help to explain the positive benefits of mindfulness for emotion regulation (Lindsay & Creswell, 2017, 2018). The first component, attention, may promote one's awareness of present‐moment emotions and emotion‐eliciting events (Boden et al., 2015; Garland & Fredrickson, 2013), which are essential elements of the emotion regulation process (Gross, 2015). Nonjudgment, the second component of mindfulness, facilitates engagement with one's emotions as individuals welcome pleasant or unpleasant emotions into their field of awareness (Lindsay & Creswell, 2017; Teper et al., 2013). Collectively, the synergistic effects of attention and nonjudgment allow for individuals to recognize emotional stimuli (Boden et al., 2015; Garland & Fredrickson, 2013) and effectively label, differentiate and/or monitor one's emotions (Creswell et al., 2007; Fogarty et al., 2015), which provides an opportunity to engage in effective emotion regulation strategies (e.g., cognitive reappraisal; Brockman et al., 2017; Curtiss et al., 2017).

Further, in a comprehensive review of mindfulness and emotion regulation, Guendelman et al. (2017) proposed a model for understanding the psychobiological benefits of trait mindfulness and mindfulness training for emotion regulation. Within this model, mindfulness among novice mindfulness practitioners may result in the “top‐down” recruitment and activation of prefrontal regulatory regions of the brain as well as emotion‐generative brain regions such as the amygdala that underlie emotion regulation (e.g., see reviews by Creswell & Lindsay, 2014; Guendelman et al., 2017). These “top‐down” regulatory processes may also be particularly relevant for adolescents who are still developing the capacity for mindfulness and emotion regulation (Jankowski & Holas, 2014; Young et al., 2019). Among long‐time mindfulness practitioners, mindfulness may instead give rise to “bottom‐up” regulatory processes that are more subconscious in nature (Guendelman et al., 2017). Taken together, both the MAT theory (Lindsay & Creswell, 2017, 2018) and the Guendelman et al. (2017) model of mindfulness support the notion that mindful attention and nonjudgment theoretically give rise to emotion regulation through top‐down and bottom‐up processes within the brain.

## EMPIRICAL EVIDENCE FOR THE CONNECTION BETWEEN MINDFULNESS AND EMOTION REGULATION

Despite conceptual overlap between mindfulness and emotion regulation (Coffey et al., 2010), cross‐sectional and experimental research suggests that mindfulness and emotion regulation are similar, but distinct processes. More specifically, mindfulness and emotion regulation exhibit overlapping characteristics as emotional identification is the first step within the extended process model of emotion regulation (Gross, 2015). Multidimensional measurements of mindfulness also include facets of emotion regulation such as nonreactivity (Baer et al., 2006). Nonetheless, mindfulness helps to stabilize one's executive attention (Morrison & Jha, 2015), which can promote greater awareness of general emotional states and emotional reactions to stimuli (Heppner et al., 2008). This attentional foundation then provides a “choice point” from which an individual can choose to engage, or not engage, in deliberate regulation (Heppner et al., 2008). In other words, attention cultivated through mindfulness conceptually precedes the selection of an emotion regulation strategy, which may be particularly relevant for adolescents who are still developing regulatory capacities (Young et al., 2019).

This distinction is further supported by research which suggests that mindfulness and emotion regulation are uniquely associated with mental health outcomes. For example, correlational studies among healthy adolescents and college students have found that mindfulness and emotion regulation, each, help to explain a unique proportion of the variance in mental health symptoms (Hambour et al., 2018) and negative affect (Vujanovic et al., 2010). Two studies that utilized atemporal meditation (Ma & Fang, 2019; Pepping et al., 2016) also found that emotion regulation difficulties help to explain the relationship between mindfulness and mental health among adolescents. Although longitudinal investigations of the relationship between mindfulness and emotion regulation among adolescents are limited, the existing correlational research provides preliminary support for the notion that self‐reported mindfulness and emotion regulation are overlapping, but distinct constructs that contribute to adolescent mental health.

Experimental studies further support the notion that greater mindfulness may give rise to lower emotion regulation difficulties. One study focused on eliciting emotion regulation among healthy adults, found that having greater trait mindfulness was associated with lower late positive potential, a neural marker of lower emotional arousal and greater emotion regulation, after viewing emotion‐eliciting images (Brown et al., 2013). Similarly, during an emotion regulation task, healthy adolescents with higher trait mindfulness displayed smaller event‐related potentials, which are the neural mechanisms that help to explain emotion dysregulation, as well as greater right EEG asymmetry (relative to left), which is indicative of more adaptive emotional responding and regulation, after viewing affective images compared with adolescents with lower trait mindfulness (Deng et al., 2020, 2021). Within an experimental study focused on inducing mindfulness and eliciting emotion regulation, older adolescents who received a breath‐focused meditation compared with a control group that did not receive the meditation also exhibited more adaptive emotional processing after viewing affective pictures (Zhang et al., 2019). These findings provide further evidence that mindfulness may indeed activate top‐down regulatory prefrontal systems in the brain that support adaptive emotion regulation. For a full review on associations between mindfulness and emotion regulation, see Guendelman et al. (2017) and Roemer et al. (2015).

However, despite theoretical (Gross, 2015; Lindsay & Creswell, 2018) and experimental evidence (Brown et al., 2013; Deng et al., 2020, 2021; Zhang et al., 2019) which posits that attention to emotions precedes regulation, several gaps limit our ability to conclude that mindfulness definitively leads to improved emotion regulation among adolescents. Most theory and empirical work rely on adult samples, whereas emotion regulation and mindfulness capacities are still developing across adolescence. Although adolescents are capable of practicing mindfulness (Brown et al., 2013; Miller et al., 2024) and recent evidence suggests that mindfulness remains relatively stable from grades 9–12 (Warren et al., 2020), developmental differences between adults and adolescents (Steinberg, 2014), as well as variability in emotion regulation capabilities across adolescence (Young et al., 2019), may alter temporal relationships. For example, emotion regulation skills increase substantially across adolescence (Young et al., 2019) and it is possible that mindfulness may confer the greatest benefit for supporting internal regulation among older adolescents, who, on average, possess more advanced emotion regulation capacities. Furthermore, the lack of longitudinal evidence makes it difficult to determine causality. As such, it is essential to leverage longitudinal data that allow researchers to explore the theoretical argument that mindfulness gives rise to emotion regulation among adolescents.

## IMPORTANCE OF MOMENTARY MEASUREMENTS OF MINDFULNESS AND EMOTION REGULATION

Furthermore, the reliance on cross‐sectional and retrospective reports of emotions/behaviors with between‐subjects study designs and/or laboratory experiments focused on manipulating mindfulness and emotion regulation not only poses limits to ecological validity, but it also makes it challenging to characterize momentary associations between constructs that unfold over time (Schwarz, 2007; Shiffman et al., 2008). Understanding momentary‐level processes that occur within individuals is not only essential for understanding how individuals respond to their lived environment, but this information can also provide knowledge about process‐oriented changes that can occur within individuals from moment to moment in response to real‐time stimuli (Schwarz, 2007; Shiffman et al., 2008). One approach that offers an alternative to traditional retrospective self‐report measures and laboratory experiments is ecological momentary assessment (EMA). EMA involves intensive, repeated measurements of socioemotional, cognitive, and behavioral processes in real time as individuals go about their daily lives. EMA can also help to minimize recall bias, maximize ecological validity, and support the examination of momentary‐level processes (Schwarz, 2007; Shiffman et al., 2008).

EMA studies have revealed that mindfulness and emotion regulation fluctuate from moment to moment in response to emotion‐eliciting stimuli (Bai et al., 2020; Miller et al., 2024) and help to explain processes central to mental health (Enkema et al., 2020; Shin et al., 2022). For example, mindfulness and emotion regulation reported within adolescents' daily lives diminish in the presence of momentary stressful life events (Miller et al., 2024). In addition, greater momentary reports of mindfulness are associated with less affective instability (Keng & Tong, 2016) and fewer mental health problems among adults (Enkema et al., 2020). Momentary‐level decreases in mindfulness are also associated with increases in psychological distress among adolescents (Lucas‐ et al., 2021). A meta‐analysis focused on the longitudinal and momentary‐level effects of mindfulness also revealed that EMA may provide more sensitive and valid measures of mindfulness, affect and depression when compared with outcomes that were reported retrospectively (Moore et al., 2016). Similarly, when daily diary reports of emotional expression and flexibility, which are central to emotion regulation (Aldao et al., 2015; Gross, 2014), were compared with momentary‐level reports, momentary‐level reports were significantly associated with anxiety and depression diagnoses and daily diary reports were not associated with these diagnoses (Shin et al., 2022). Taken together, mindfulness and emotion regulation are dynamic processes that unfold over time and can help to explain the development and maintenance of mental health problems, but there has yet to be an investigation of the role that mindfulness plays in affecting emotion regulation at the momentary level. Although it is possible that moments of greater mindfulness (i.e., greater within‐person mindfulness) may give rise to fewer emotion regulation difficulties given the consistent between‐subjects evidence for negative associations between mindfulness and emotion regulation difficulties (Ma & Fang, 2019; Pepping et al., 2016) as well as EMA evidence that momentary mindfulness is inversely associated with affective instability (Keng & Tong, 2016), this hypothesis warrants further investigation.

## MODERATING EFFECTS OF MENTAL HEALTH

Mindfulness and emotion regulation have both been extensively explored as mediators and moderators of mental health processes (e.g., Chen & Cheung, 2021; Cortazar & Calvete, 2019; Ma & Fang, 2019; Pepping et al., 2016) and various treatment studies (Chiesa et al., 2014; Keng et al., 2012; D. Zhang et al., 2021). However, few have investigated if the relationship between mindfulness and emotion regulation varies as a function of an individual's mental health. Internalizing and externalizing symptoms, PTSD symptoms and attention problems are some of the most common and highly prevalent mental health challenges that US adolescents face (Bitsko et al., 2018; Whitney & Peterson, 2019), especially in the wake of the COVID‐19 pandemic (Chavira et al., 2022). Unfortunately, individuals with greater mental health problems tend to experience lower mindfulness (Cortazar & Calvete, 2019; Greco et al., 2011; Heppner et al., 2008; Royuela‐Colomer et al., 2021) and greater emotion regulation difficulties (Aldao et al., 2010; Sheppes et al., 2015) as well as challenges with engaging in mental health treatment protocols (Coles et al., 2004; Lewis et al., 2020; Wright et al., 2021). Interestingly, among adolescents and adults with mental health challenges, there are reports of mindfulness increasing distress (Didonna & Gonzalez, 2009; Forner, 2019; Hall, 2020). Similarly, in the presence of only mindful attention, awareness of one's thoughts and emotions may give rise to emotional reactivity and emotion dysregulation (Lindsay & Creswell, 2017). Alternatively, the mindfulness stress buffering hypothesis posits that mindfulness may be most beneficial under conditions of high stress (Creswell & Lindsay, 2014), such that populations who experience a high degree of stress and consequently greater mental health problems, may benefit most from mindfulness and mindfulness‐based interventions. In addition, within a systematic review focused on meta‐analyses of randomized controlled trials for mindfulness‐based interventions, the largest treatment effects were observed among populations with high levels of anxiety, depression, and psychopathology (Enkema et al., 2020). However, this systematic review did not review trait or momentary‐level benefits of mindfulness for clinical or nonclinical populations. Taken together, mental health likely influences the relationship between mindfulness and emotion regulation, but the direction of the effects is unclear. To determine the clinical utility of mindfulness for emotion regulation among clinical populations, it is essential to investigate the moderating effects of mental health on this relationship.

## CURRENT STUDY

The aim of the present study was to investigate the benefits of between and within‐person mindfulness for emotion regulation among adolescents and to explore the moderating effects of mental health symptoms (i.e., internalizing, externalizing, PTSD symptoms, and attention problems) on these relationships. In line with existing literature (Brown et al., 2013; Ma & Fang, 2019; Pepping et al., 2016; Zhang et al., 2019), we hypothesized that individuals with higher trait levels of mindfulness would report fewer emotion regulation difficulties. Similarly, we expected moments of greater than average levels of mindfulness to be associated with fewer emotion regulation difficulties within the same moment that mindfulness was reported (time 1) and within the subsequent moment (time 2). In other words, between and within‐subjects mindfulness would be inversely associated with concurrent and prospective reports of emotion regulation difficulties. In addition, we hypothesized that individuals with greater mental health challenges would experience a diminished effect between mindfulness and emotion regulation. Although existing research suggests that individuals with greater mental health problems may have lower levels of mindfulness and greater emotion regulation difficulties (Aldao et al., 2010; Cortazar & Calvete, 2019; Greco et al., 2011; Heppner et al., 2008; Royuela‐Colomer et al., 2021; Sheppes et al., 2015), which may help to explain these potentially diminished effects, the benefits of mindfulness for clinical populations are mixed (Didonna & Gonzalez, 2009; Enkema et al., 2020; Forner, 2019; Hall, 2020); therefore, this aim is exploratory.

## METHOD

Participants were adolescents participating in a larger randomized control trial investigating the benefits of a mindfulness‐based intervention for mental and behavioral health. Inclusion criteria included involvement in a site‐based mentoring program, Campus Connections, being between the ages of 10–18 years old and being English‐speaking because the mindfulness intervention was only offered in English. Youth were referred to the program by parents, guardians or community organizations for inhibiting environmental and individual risk (e.g., Department of Human Services/juvenile‐justice involvement, behavioral/emotional problems). Although a mental health diagnosis was not an inclusion criterion for this study, 56% of adolescents (*n* = 44) had been diagnosed with a mental health disorder by a medical professional and 50% (*n* = 40) experienced clinically elevated PTSD symptoms with a score of 16 or greater on the Child PTSD Symptom Scale (Foa et al., 2001). Given these characteristics, this sample may be considered clinical in nature. A total of 81 adolescents participated in the research study and completed at least one EMA survey at baseline. On average, adolescents were 13.75 years old (SD = 2.17 years); 56% identified as a boy/male (*n* = 45), 37% identified as a girl/female (*n* = 30), and 7% identified as another gender (*n* = 6). A total of 57% of adolescents identified as non‐Hispanic White (*n* = 46); 24% identified as Hispanic/Latino (*n* = 19); 7% identified as native American (*n* = 6); 7% identified as more than one race (*n* = 6); and 5% identified as Asian or Pacific Islander or Black/African American (*n* = 4; categories combined to protect confidentiality).

Of the parents/guardians that provided demographic information (*n* = 73), 50% (*n* = 37) reported making less than $40,000 a year, and the median household income ranged from $40,000 to $59,999. Eleven percent (*n* = 8) of parents/guardians reported that they did not complete high school, 15% completed high school (*n* = 12), 20% (*n* = 16) reported having a GED or completing some college, 20% (*n* = 16) had an associate's degree, 14% (*n* = 11) had a bachelor's degree, and 12% (*n* = 10) had a graduate‐level degree. In addition, 55% of parents/guardians placed themselves on the bottom five rungs of the ladder on the MacArthur Scale of Subjective Social Status (Adler et al., 2000), which indicated that they believed that they were worse off compared with other people in the United States.

### Procedures

During a baseline visit, before the youth participated in the mentoring program, the adolescents and their parents/guardians provided informed consent and assent, respectively, and completed a survey designed to assess mental health problems, behavioral problems and demographic characteristics. Adolescents also installed the EMA data collection application, TigerAware (Morrison et al., 2018), onto their smartphones and received training in how to complete EMA surveys. During this training, teens watched a short video on what the EMA questions looked like, what each question meant and how to answer and submit each survey. The EMA surveys began the day after the baseline visit and took approximately 5 min to complete. Adolescents received three EMA surveys per day for a total of 7 days, and each time they were signaled to complete a survey, they had up to 30 min to answer before the survey expired. Depending upon how early the participant started and ended school, weekday surveys arrived at random times between after‐school hours of 3:00 pm and 9:00 pm, or between before‐school hours of 7:00 am and 8:15 am and after‐school hours of 4:15 pm and 9:00 pm. On the weekends, surveys arrived at random times between 9:00 am and 9:00 pm. Participants were paid $1 for each survey completed and if they answered at least 76% of all surveys (i.e., 16 out of 21 surveys), they received a $5 bonus. All research procedures were approved by the Institutional Review Board at Colorado State University (Protocol #2008).

### Measures

#### Mindfulness

Two key dimensions of mindfulness were assessed using EMA: mindful attention/awareness and mindful nonjudgment. Items were drawn from reliable and valid mindfulness questionnaires and adapted to correspond to the momentary assessment timeframe. Five items were adapted from the Mindful Attention and Awareness Scale (Brown & Ryan, 2003) and were used to assess mindful attention/awareness. Participants rated the extent to which each item (e.g., “I am preoccupied with the past or future”) was currently consistent with their experience (i.e., at the moment of data collection) on a 7‐point Likert scale from 1 (*not at all*) to 7 (*very much*). All items were reverse scored and averaged with higher scores indicating greater mindful attention (Ω_b_ = .93; Ω_w_ = .78). Participants also completed one item adapted from the Self‐Compassion Scale for Children‐Short Form (Sutton et al., 2018) to assess mindful nonjudgment. Participants rated how much “I feel disapproving and judgmental of the things I don't like about myself or my flaw” currently applied to them on a 7‐point Likert scale from 1 (*not at all*) to 7 (*very much*). This item was reverse scored; higher scores indicate greater levels of mindful nonjudgment.

#### Emotion regulation difficulties

Participants completed four items from the State‐Difficulties in Emotion Regulation Scale (S‐DERS; Gratz & Roemer, 2004; Lavender et al., 2017) via EMA, which is a state‐oriented measure of emotion regulation difficulties that is based on the original trait‐oriented DERS (Gratz & Roemer, 2004). Items with the highest factor loadings on each subscale (i.e., nonacceptance, awareness, modulate, and clarity) from the S‐DERS were selected (Lavender et al., 2017). Participants responded to how much a statement (e.g., “I feel embarrassed for feeling how I feel”) currently applied to their emotional experiences on a scale of 1 (*not at all*) to 7 (*completely*). When calculating scale reliability, the awareness item had a low factor loading (0.08), and therefore, was not included in the analysis. Scores on the three remaining items (“I feel embarrassed for feeling how I feel,” “I am having a hard time controlling my behaviors,” and “I am confused about how I feel”) were averaged with higher scores indicating greater difficulties with emotion regulation (Ω_b_ = .90; Ω_w_ = .66).

#### PTSD symptoms

During a baseline study visit, participants completed the 17‐item Child PTSD Symptom Scale (Foa et al., 2001). Adolescents considered a distressing event and reported on how it affected them in the previous 2 weeks using a 4‐point scale (0 = not at all to 3 = almost always). A total summed score was calculated with higher values representing greater PTSD symptoms (Cronbach's α = .94). PTSD symptoms were then standardized by subtracting the mean from each raw score and dividing by the standard deviation, allowing for comparison across different measures.

#### Mental health symptoms

During a baseline study visit, participants completed the 19‐item Brief Problem Monitor (BPM). Participants rated how much an item (e.g., “I argue a lot”) described them now or within the past 7 days using a 3‐point scale (0 = not true to 2 = very true). The BPM consists of three subscales, which measure internalizing symptoms (α = .87), externalizing symptoms (α = .71) and attention problems (α = .68). The scores for each subscale were summed separately with higher scores indicating greater symptoms. All mental health measures were then standardized to have a mean of 0 and a standard deviation of 1 to allow for comparison across measures.

## DATA ANALYSIS

Prior to data analysis, variables were checked for normality and all variables were found to be significantly skewed. To account for nonnormality, a Bayes estimator was used within all analyses, which is robust to nonnormality (Asparouhov & Muthén, 2010). In addition, the outcome variable, difficulties with emotion regulation, had a strong floor effect; therefore, a two‐part censor‐inflated model was applied to attenuate potential bias (Muthén et al., 2025). Next, intraclass correlations (ICCs) were investigated within an intercept only, unconditional random effects model to determine if multilevel modeling was appropriate. ICCs describe the proportion of variance in a variable that can be explained by the grouping variable, in this case, by participant. The ICCs were all above 0.05 and the assumption of independence was violated (Kreft & De Leeuw, 1998; Table S1). In line with recommendations by McNeish and Hamaker (2020), stationarity was investigated by regressing emotion regulation difficulties, specified as a below‐inflated censored variable, on time. Neither the continuous (*b* = −.009, *p* = .20) nor the censored‐inflated component of the model (*b* = .014, *p* = .14) showed significant associations with time, suggesting that the assumption of stationarity was met.

To test study hypotheses and to account for nesting with the data, we ran a two‐part, two‐level dynamic structural equation model (DSEM) with random intercepts and slopes. DSEM is extremely flexible and combines structural equation modeling, multilevel modeling, and time series analysis to investigate intensive repeated measurements across multiple levels of data, including at the momentary level (Asparouhov et al., 2018; McNeish & Hamaker, 2020). Two‐part models split the outcome into two observed variables; one variable represents the presence (i.e., not at the floor) or absence (i.e., at the floor) of emotion regulation difficulties and the other is a continuous variable that represents the degree of difficulty for those not at the floor value (Muthén et al., 2025). Momentary assessment was specified as level one (within‐person) and individual was specified as level two (between‐person), which is in line with past EMA research (Curran, 2003). We also accounted for the autoregressive (AR 1) relationship between emotion regulation difficulties reported within the current moment (T) and the previous moment (T‐1). Analyses of processes that occurred within the same moment (concurrently; T) and the next moment (i.e., mindfulness reported in T‐1 and prospective associations with emotion regulation in the current moment [T]) were analyzed within one model. Within‐person variables were automatically disaggregated to represent deviations from each person's average while between‐person variables represent an individual's overall average (Asparouhov & Muthén, 2019). In addition, 95% Bayesian Credible Intervals (95% CI) were calculated to determine the true value of a parameter with 95% probability. We applied noninformative priors, which resulted in estimates that converge with those that would have been obtained under maximum likelihood estimation (Muthén et al., 2025); therefore, we interpreted 95% CIs that did not contain zero to represent statistical significance as is typical in frequentist analysis. Missing data were handled using Bayesian estimation (Enders, 2023). A random walk algorithm with 50,000 iterations was also specified to establish model stability. Potential Scale Reduction (PSR) for all models was below 1.03 by 50,000 iterations, which suggests adequate model convergence (Gelman et al., 1995). Effect sizes were determined by evaluating standardized coefficients with 0.1 representing a small, 0.3 representing a medium and .05 representing large effects (Cohen, 2013).

The random coefficient prediction (RCP) method (Preacher et al., 2016) was used to explore moderation hypotheses. The RCP method involves creating a random slope between mindfulness and emotion regulation difficulties at the within‐person level, which is then predicted by the between‐person moderator variable. This method allows for 2(1 × 1) moderation, which is also known as a cross‐level interaction. All moderators were included within one model. To visualize significant moderation results, the random slopes were exported from Mplus and plotted against the predictor variable in R using “ggplot2” (Wickham & Sievert, 2009). The random slope was placed on the *y*‐axis and the between‐level moderator was placed on the *x*‐axis. Based on the power tables outlined by Arend and Schäfer (2019) for multilevel models, we had adequate power to detect small effects (see Arend & Schäfer, 2019 for more information). All DSEM analyses were conducted within Mplus version 8.9 (Muthén & Muthén, 2017). Data and code are available (https://osf.io/8xeyg/?view_only=f7a96a4a59ea4e6ba8b3eb7e044cbeda). All study hypotheses and the analysis plan were preregistered on the Open Science Framework with the exception of attention problems. Given that attention problems are a key dimension of the BPM, they were included in analyses after the study was preregistered.

## RESULTS

Eighty‐one participants provided 1186 total EMA data points. However, two individuals were removed from the sample because their estimates exhibited nonstationarity. As such, the remaining 1158 EMA surveys from 79 participants were used within study analyses. The average cluster size was 14.64, which indicates that the average number of surveys completed was ~ 70% (14 out of 21) of total possible surveys. Within a day, adolescents completed 2.15 EMA surveys (*SD* = 0.79). Rates of compliance were similar to other EMA studies with children and adolescents (Heron et al., 2017; Wen et al., 2017). The number of completed EMA surveys was not associated with participant age, race/ethnicity, income, or gender identity (*ps* > .59).

### Concurrent associations

**FIGURE 1:**
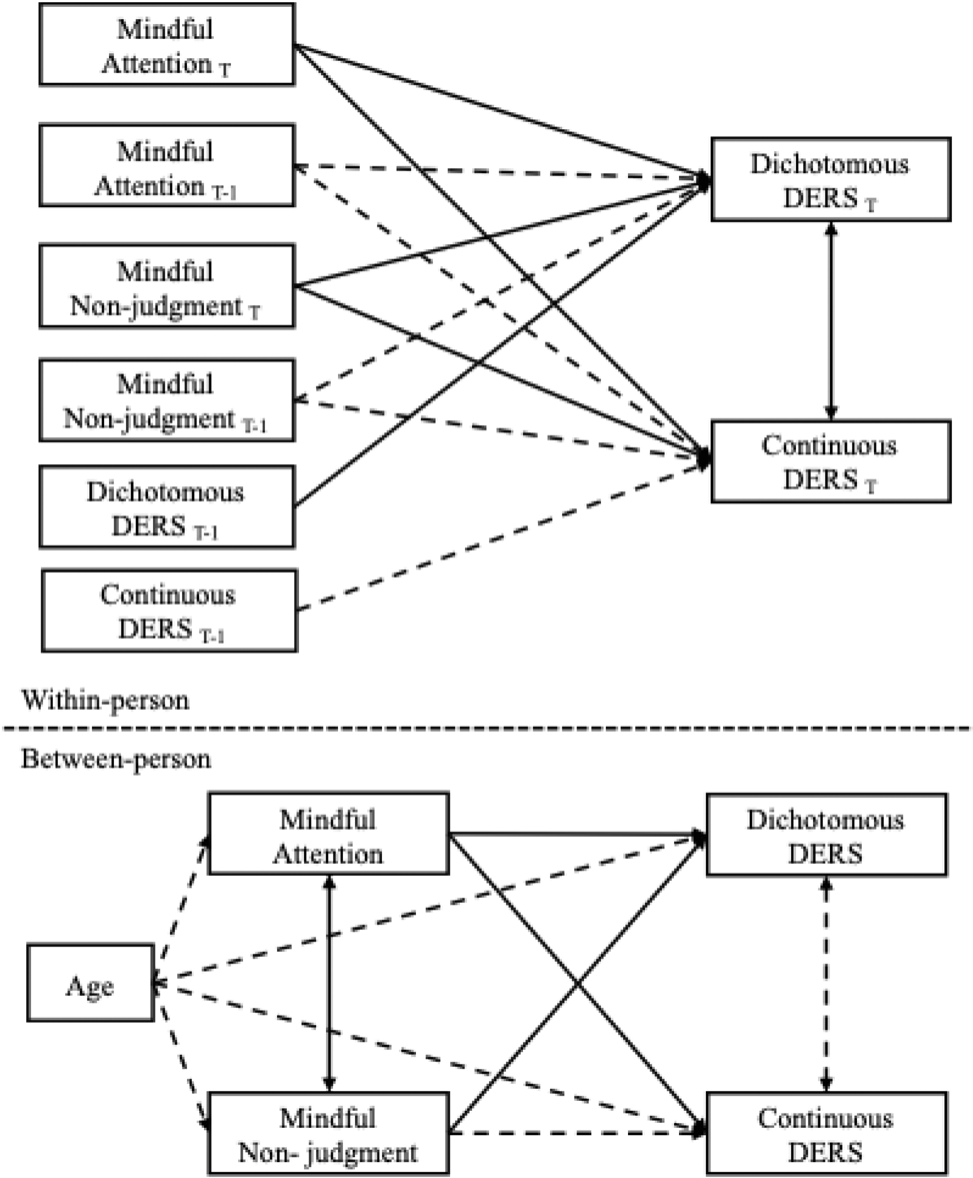
Concurrent and prospective associations between mindfulness and difficulties with emotion regulation. T represents concurrent associations and T‐1 represents prospective associations. Solid lines present significant paths, while dotted lines represent nonsignificant paths.

At the momentary (within‐person) level, concurrent mindful attention and mindful nonjudgment were both associated with a lower likelihood of reporting emotion regulation difficulties in the logistic inflation portion of the censor‐inflated paths, after controlling for the between‐subjects effect of age on mindfulness and emotion regulation (Table 1; Figure 1). Similarly, in the continuous portion of the censor‐inflated path when emotion regulation difficulties were modeled continuously, higher mindful attention and mindful nonjudgment were associated with fewer emotion regulation difficulties. Standardized coefficients suggest that the relationships between concurrent mindfulness and emotion regulation difficulties were small to medium in size. This collectively suggests that when adolescents reported being more mindful than usual in a given moment, they also tended to report fewer difficulties regulating their emotions at that same time.

**TABLE 1:** Standardized and unstandardized within‐ and between‐subjects regression coefficients of concurrent and prospective associations.

	Standardized effects			Unstandardized effects			
	*B*	SE	95% CI	*b*	SE	95% CI	OR [95% CI]
Within‐subjects effects							
DERS Dichotomous							
Mindful Attention	−.19	0.04	[−0.26, −0.12]	−.08	0.02	[−0.11, −0.04]	0.93 [0.90, 0.96]
Mindful Attention T‐1	.02	0.05	[−0.09, 0.11]	.01	0.02	[−0.03, 0.05]	1.01 [0.97, 1.05]
Mindful Nonjudgment	−.21	0.04	[−0.27, −0.14]	−.06	0.02	[−0.10, −0.03]	0.94 [0.91, 0.97]
Mindful Nonjudgment T‐1	−.00	0.05	[−0.10, 0.09]	−.00	0.01	[−0.03, 0.03]	1.00 [0.97, 1.03]
DERS Dichotomous T‐1	.11	0.04	[0.03, 0.20]	.12	0.04	[0.03, 0.21]	1.13 [1.03, 1.23]
DERS Continuous							
Mindful Attention	−.38	0.05	[−0.48, −0.29]	−.01	0.06	[−0.12, 0.10]	—
Mindful Attention T‐1	−.01	0.06	[−0.12, 0.10]	−.02	0.06	[−0.14, 0.10]	—
Mindful Nonjudgment	−.18	0.04	[−0.26, −0.10]	−.13	0.1	[−0.34, 0.05]	—
Mindful Nonjudgment T‐1	−.00	0.06	[−0.13, 0.10]	−.13	0.1	[−0.34, 0.05]	—
DERS Continuous T‐1	.12	0.06	[−0.02, 0.23]	.11	0.04	[−0.01, 0.22]	—
DERS Continuous with DERS Dichotomous	.2	0.08	[0.09, 0.41]	.08	0.05	[−0.01, 0.21]	—
Between‐Subjects Effects							
DERS Dichotomous							
Mindful Attention	−.31	0.15	[−0.59, −0.00]	−.09	0.05	[−0.18, −0.00]	0.91 [0.84, 1.00]
Mindful Nonjudgment	−.48	0.14	[−0.70, −0.17]	−.10	0.03	[−0.16, −0.04]	0.91 [0.85, 0.96]
Age	−.13	0.1	[−0.32, 0.07]	−.02	0.02	[−0.05, 0.01]	0.98 [0.95, 1.01]
DERS Continuous							
Mindful Attention	−.72	0.09	[−0.85, −0.49]	−.72	0.13	[−0.98, −0.47]	—
Mindful Nonjudgment	−.19	0.14	[−0.46, 0.07]	−.13	0.1	[−0.34, 0.05]	—
Age	.06	0.08	[−0.10, 0.22]	.03	0.05	[−0.06, 0.12]	—
Mindful Attention							
Age	−.19	0.11	[−0.39, 0.04]	−.10	0.06	[−0.22, 0.02]	—
Mindful Nonjudgment							
Age	−.17	0.11	[−0.37, 0.05]	−.13	0.09	[−0.30, 0.04]	—
DERS Continuous with DERS Dichotomous	.39	0.19	[−0.03, 0.71]	.08	0.05	[−0.01, 0.21]	—

*Note*: DERS stands for difficulties with emotion regulation. Odds ratios (ORs) were provided for all dichotomous outcomes by exponentiating the unstandardized regression coefficients. T‐1 represents prospective associations.

At the individual (between‐person) level, mindfulness was consistently associated with fewer difficulties with emotion regulation. Specifically, adolescents who, on average, reported higher mindful attention and mindful nonjudgment were less likely to report the presence of emotion regulation difficulties in the logistic portion of the censor‐inflated paths after accounting for age. In the continuous part of the censor‐inflated path, when emotion regulation was examined on a continuous scale (capturing degree of difficulty), higher mindful attention was also associated with fewer difficulties. Taken together, these results suggest that mindfulness is linked both to a lower likelihood of experiencing emotion regulation problems, and to experiencing fewer difficulties when such problems are present.

### Prospective associations

After accounting for concurrent associations and the autoregressive effect between T and T‐1 emotion regulation difficulties, mindfulness reported in one moment did not predict subsequent emotion regulation. Neither within‐person mindful attention nor mindful nonjudgment had prospective effects on the presence of emotion regulation difficulties in the logistic portion of the model. Likewise, when emotion regulation was examined on a continuous scale, mindful attention and mindful nonjudgment were not associated with next moment emotion regulation difficulties.

### Moderation of concurrent and prospective associations

**FIGURE 2:**
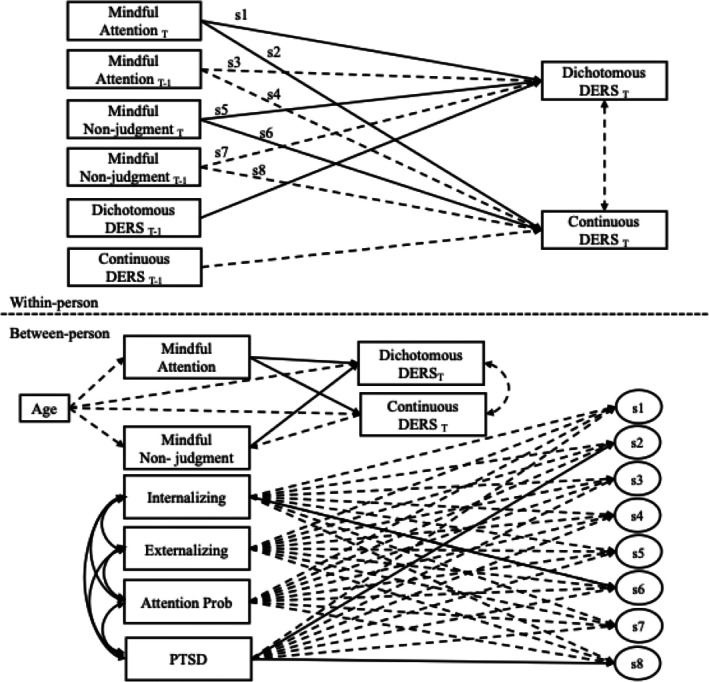
Moderation of concurrent and prospective random slopes. T represents concurrent associations and T‐1 represents prospective associations. Solid lines present significant paths and dotted lines represent nonsignificant paths.

**FIGURE 3:**
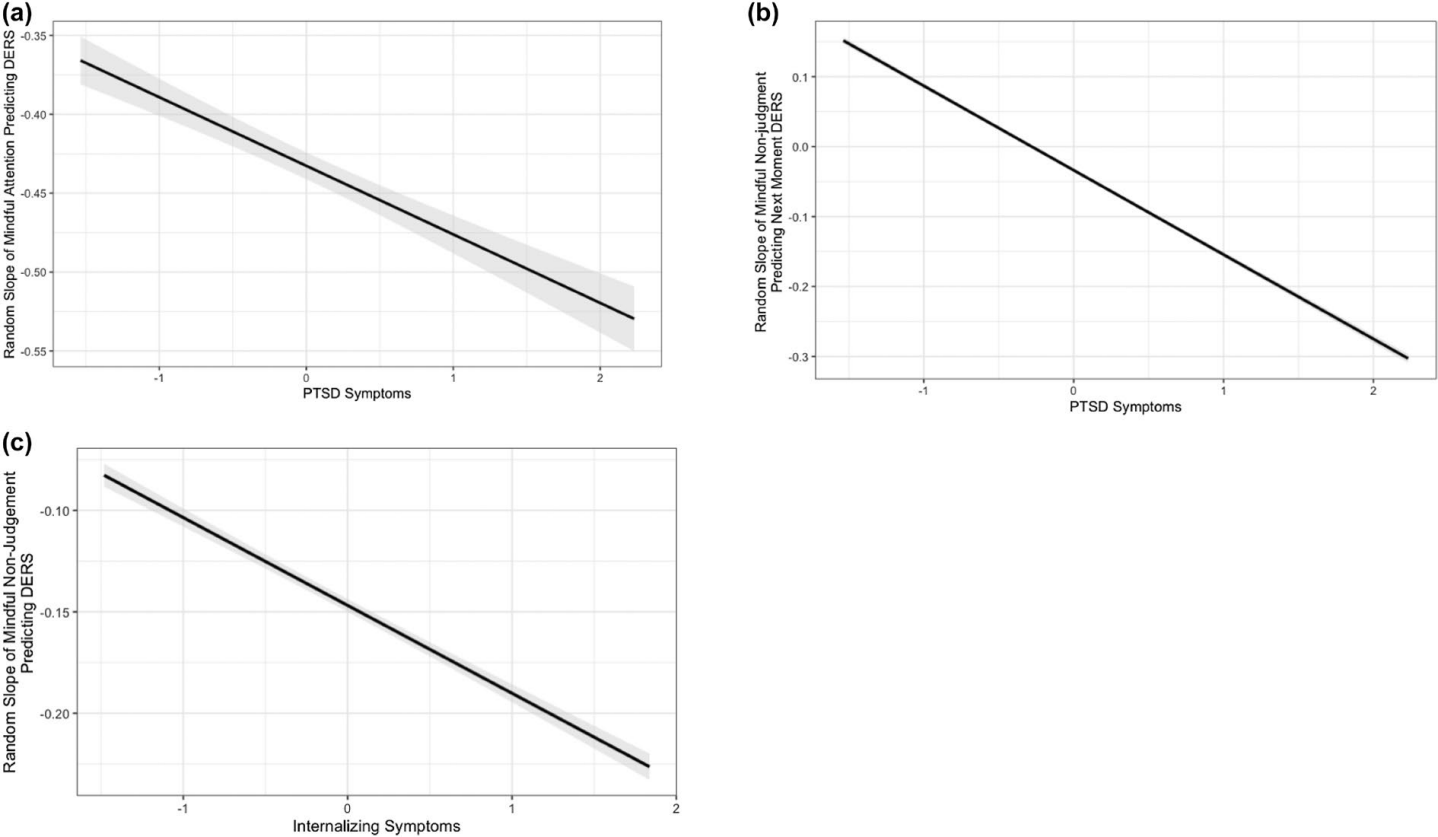
PTSD and internalizing symptoms as moderators of random slopes. (a) Represents PTSD symptoms as a moderator of the mindful attention and emotion regulation difficulties (concurrent) relationship. (b) Represents PTSD symptoms as a moderator of the prospective relationship between mindful nonjudgment and emotion regulation difficulties relationship. (c) Represents internalizing symptoms as a moderator of the mindful nonjudgment and emotion regulation difficulties (concurrent) relationship. Of note, the random slopes are all negative.

After controlling for age, PTSD symptoms moderated both concurrent and prospective associations between mindfulness and continuously measured emotion regulation difficulties. Concurrently, PTSD symptoms had a medium‐sized effect on the relationship between mindful attention and emotion regulation difficulties (*B* = −0.60, *SE* = 0.15, 95% CI [−0.62, −0.03]; Figure 2). Adolescents with higher PTSD symptoms experienced a steeper slope between mindful attention and difficulties with emotion regulation (Figure 3a). This suggests that those with higher PTSD symptoms experienced fewer difficulties with emotion regulation in moments of greater than average levels of mindful attention. In contrast, adolescents with fewer PTSD symptoms experienced a more blunted random slope which suggests that mindfulness was still associated with fewer difficulties, but the effect was smaller. Prospectively, PTSD symptoms also had a large effect on the association between mindful nonjudgment and subsequent emotion regulation difficulties (*B* = −.77 *SE* = 0.27, 95% CI [−1.21, −0.18]). More specifically, adolescents with higher PTSD symptoms who reported greater mindful nonjudgment in one moment were more likely to experience fewer emotion regulation difficulties later in the day (Figure 3b).

Internalizing symptoms also moderated concurrent associations. There was a negative medium‐sized effect of internalizing symptoms on the association between mindful nonjudgment and difficulties with emotion regulation (*B* = −.51, *SE* = 0.24, 95% CI [−0.95, −0.02]). Those with greater internalizing symptoms experienced a steeper slope between mindful nonjudgment and difficulties with emotion regulation (Figure 3c). In other words, the relationship between mindful nonjudgment and difficulties with emotion regulation was stronger for those with greater internalizing symptoms, while the relationship between mindful nonjudgment and difficulties with emotion regulation was weaker for those with fewer internalizing symptoms. Practically, this suggests that moments of greater mindfulness may be particularly impactful for adolescents experiencing PTSD and internalizing symptoms. There were no other significant moderating effects of PTSD symptoms, internalizing, externalizing or attention problems on concurrent or prospective associations between mindfulness and emotion regulation.

## DISCUSSION

The goal of the current study was to characterize the benefits of between‐person and within‐person mindfulness for emotion regulation among adolescents and to explore the moderating effects of mental health symptoms (i.e., internalizing, externalizing, PTSD symptoms, and attention problems) on the momentary‐level relationship between mindfulness and emotion regulation. Our findings indicate that greater within‐person mindfulness was associated with lower emotion regulation concurrently, but not prospectively from one moment to the next. We also discovered that PTSD and internalizing symptoms moderated concurrent associations between mindfulness and emotion regulation such that individuals with higher mental health symptoms displayed fewer emotion regulation difficulties during moments of above‐average mindfulness. In addition, we observed that prospective associations between mindful nonjudgment and emotion regulation difficulties were most beneficial for individuals with higher PTSD symptoms. These findings are meaningful as they not only provide support to the MAT theory, but they also shed light on the clinical utility of momentary mindfulness for emotion regulation for adolescents with greater PTSD and internalizing symptoms.

In line with our hypotheses, the MAT theory (Lindsay & Creswell, 2017) and between‐subjects research (Brown et al., 2013; Ma & Fang, 2019; Pepping et al., 2016), moments of greater than average mindful attention and nonjudgment were associated with lower emotion regulation difficulties. Individuals with higher average levels of mindful attention and nonjudgment also experienced fewer emotion regulation difficulties overall. These momentary‐level findings highlight how mindfulness can fluctuate from moment to moment and confer benefits in everyday life, while the trait‐level results suggest that cultivating higher levels of mindfulness over time may be associated with more consistent emotion regulation.

These momentary‐level relationships were also particularly meaningful for individuals with elevated PTSD and internalizing symptoms. Among adolescents with higher PTSD symptoms compared with lower PTSD symptoms, there was a stronger association between concurrent mindful attention and lower emotion regulation difficulties as well as between mindful nonjudgment and prospective emotion regulation difficulties. Similarly, the relationship between mindful nonjudgment and emotion regulation was strongest among adolescents with higher internalizing symptoms. These results may be explained, in part, by the mindfulness stress buffering hypotheses (Creswell & Lindsay, 2014), which posits that individuals in high‐stress environments who consequently experience greater mental health challenges, may derive the most robust benefits of mindfulness. These study results are also supported by the fact that induced mindfulness can promote more adaptive emotion regulation (Zhang et al., 2019). Interestingly, however, these findings do not support the idea that mindful attention and mindful nonjudgment are both necessary to support emotional well‐being (Lindsay & Creswell, 2017). Instead, they highlight how the two dimensions of mindfulness may provide distinct benefits to emotion regulation depending on the type of mental health symptomatology. For adolescents with PTSD symptoms, greater awareness of the present moment may help to facilitate the attentional deployment phase within the emotion regulation process whereby increased attention may enhance opportunities to engage in cognitive change and more adaptive behavioral responses (Gross, 2015). Conversely, given the strong associations between shame/self‐judgment and PTSD symptoms (Seah et al., 2023) and due to the fact that the capacities for emotion regulation are still developing during adolescence (Young et al., 2019), it might be challenging for adolescents with PTSD to regulate their emotions while practicing nonjudgment. Instead, mindful nonjudgment practiced earlier in the day may create opportunities for acceptance and emotion regulation later in the day. In contrast, for youth with internalizing symptoms such as anxiety and depression, mindful nonjudgment may facilitate the selection of more adaptive regulation strategies within the same moment (Lindsay & Creswell, 2017). Meanwhile, mindful attention may exacerbate emotional reactivity for adolescents with internalizing symptoms (Lindsay & Creswell, 2017). Going forward, it may be helpful to experimentally manipulate the two dimensions of mindfulness to better isolate their unique benefits for different mental health symptoms. Collectively, however, our results highlight how mindfulness might be a potentially accessible strategy for supporting emotion regulation within daily life among adolescents who may be most vulnerable to emotional distress.

Contrary to our hypotheses, moments of greater than average levels of mindfulness did not give rise to significantly fewer emotion regulation difficulties within the next moment when the entire sample was considered. Instead, only concurrent relationships were significant. This finding suggests that heightened present‐moment awareness and nonjudgmental appraisals may not exert lasting effects beyond the immediate moment in which they occur for youth as a whole. Although the lack of prospective findings makes it challenging to assess temporal claims, these results are in line with the fundamental definition of mindfulness as a present‐moment state (Kabat‐Zinn, 1994). As such, mindfulness may primarily promote more adaptive emotion regulation in real time. Alternatively, however, it is also possible that within‐person mindfulness does not give rise to fewer emotion regulation difficulties among youth without mental health difficulties. Although this is unlikely given experimental evidence that induced mindfulness does indeed support “top‐down” and “bottom‐up” regulatory processes (Brown et al., 2013; Deng et al., 2020, 2021; Zhang et al., 2019), this potential explanation warrants additional exploration. In addition, the lack of prospective findings may be due to the fact that only three EMA surveys occurred each day. There were often several hours in between each EMA survey, and this may have limited our ability to investigate the more short‐term dynamics between mindfulness and emotion regulation. Within future research, it will be important to incorporate more than three intensive repeated measurements within a day to more definitively characterize the temporal, momentary‐level relationship between mindfulness and emotion regulation difficulties.

Similarly, we had hypothesized that externalizing symptoms and attention problems would moderate the relationship between mindfulness and emotion regulation difficulties, but neither of these mental health symptoms emerged as significant moderators. This tentatively suggests that the momentary‐level relationships between mindfulness and emotion regulation do not change as a function of an adolescent's externalizing or attention problems. In other words, adolescents may benefit from momentary mindfulness regardless of their externalizing symptoms or attention problems. This potential explanation is partially supported by research that suggests that greater between‐person mindfulness is associated with fewer emotion regulation difficulties in nonclinical samples experiencing varying levels of internalizing and externalizing symptoms (Ma & Fang, 2019; Pepping et al., 2016). Alternatively, given that this sample did not specifically include adolescents with clinical mental health diagnoses, it is also possible that adolescents with clinically elevated levels of externalizing symptoms and attention problems may experience a different relationship between momentary mindfulness and emotion regulation. Notably, there is limited research on the relationship between trait and state mindfulness and emotion regulation within clinical samples. Future work may benefit from recruiting adolescents with diagnosed mental health conditions to more conclusively determine whether mindfulness provides consistent benefits for emotion regulation across varying levels of mental health symptoms.

### Clinical implications

Results of this study carry several implications for treatment and practice. First, these results highlight that individuals experiencing emotion regulation difficulties within daily life may benefit from increases in present‐moment attention and nonjudgmental awareness. Clinicians and therapists might consider guiding clients through mindfulness practices during moments of emotional difficulty as greater momentary mindfulness may help to support emotion regulation in real time. Second, this research may inform the development of just‐in‐time (JIT) interventions, which are interventions that can occur within daily life outside of the research lab or therapy session. Our findings suggest that greater than average levels of mindfulness may not always be beneficial for teens' moments or hours later. Instead, teens may need accessible training in mindfulness at the very moment that they are experiencing emotion regulation difficulties. In other words, these results highlight the need for JIT interventions that extend beyond a 50‐min therapy session. In addition, single‐session JIT interventions may have limited effectiveness for long‐term or ongoing emotional difficulties, and it may be beneficial to offer or incorporate multiple JIT interventions throughout the day. This research also highlights the importance of tailoring mindfulness‐based interventions for adolescents with specific mental health symptoms. While mindful attention may be particularly beneficial for adolescents with PTSD symptoms, the prospective associations for mindful nonjudgment suggest that providing additional supports for cultivating nonjudgment could help adolescents with PTSD experience benefits to emotion regulation both in the same moment and later in the day. In contrast, adolescents with broad‐based internalizing symptoms may benefit from additional supports when practicing mindful attention. Within future research, scientists and researchers should explore how trainings in specific dimensions of mindfulness might produce unique benefits for individuals with differing mental health concerns.

### Limitations

There are several important limitations to consider when interpreting study results. First, EMA surveys only occurred three times a day and additional short‐term effects of mindfulness may have been missed. Although three EMA surveys are typically used to assess momentary‐level relationships between emotional and behavioral processes (Heron et al., 2017; Wen et al., 2017), it may be helpful to incorporate additional EMA surveys within future research. Second, this study only investigated emotion regulation difficulties as opposed to emotion regulation strategies. To more completely understand how mindfulness may help to support emotion regulation processes, it will be important to measure the emotion regulation strategies that adolescents selected to use within moments of greater mindfulness. Third, this sample only included adolescents exposed to high levels of chronic stress and results may not be generalizable to samples of adolescents not exposed to fewer stressors.

## CONCLUSIONS

This study contributes meaningful knowledge about the momentary‐level relationships between mindfulness and emotion regulation difficulties. This study also helps to shed light on the clinical utility of mindfulness for emotion regulation among adolescents with mental health difficulties. Results suggest that moments of greater than average levels of mindfulness are associated with fewer emotion regulation difficulties, but these relationships may not extend prospectively for all youth. Individuals with greater PTSD symptoms may benefit from both dimensions of mindfulness, but the effects of mindful nonjudgment may not be immediate. For adolescents with greater internalizing symptoms, on the other hand, mindful nonjudgment may be most beneficial when attempting to reduce current moment emotion regulation difficulties. Going forward, it will be important to investigate how different dimensions of mindfulness may yield differential benefits depending on an individual's mental health concern. It may also be helpful to incorporate additional EMA measurements to explore short‐term temporal dynamics between mindfulness and emotion regulation. Collectively, these findings provide support for the notion that within‐person mindfulness may help to reduce emotion regulation difficulties in real‐time among adolescents experiencing mental health difficulties.

## AUTHOR CONTRIBUTIONS

**Reagan L. Miller‐Chagnon:** Conceptualization; investigation; funding acquisition; writing – original draft; methodology; visualization; writing – review and editing; formal analysis; data curation; project administration. **Shelley A. Haddock:** Funding acquisition; writing – original draft; writing – review and editing; supervision; project administration; resources. **Toni S. Zimmerman**: Funding acquisition; writing – original draft; writing– review and editing; supervision; project administration; resources. **Mark A. Prince:** Writing – original draft; funding acquisition; validation; supervision; data curation; software; formal analysis; writing – review and editing.

## FUNDING INFORMATION

Funding was provided by the National Center for Complementary and Integrative Health (F31AT011642) and the Colorado Agricultural Experiment Station/National Institute of Food and Agriculture (COL00789).

## CONFLICT OF INTEREST STATEMENT

None to report.

## ETHICAL APPROVALS

All research procedures were approved by the Institutional Review Board at Colorado State University (CSU; Protocol # 2008) on June 14, 2021.

## PATIENT CONSENT

Adolescents and their parents/guardians provided informed consent and assent, respectively, prior to the completion of any study procedures with a trained member of the research team.

## Supporting information


**Table S1.** Descriptive statistics for key study variables.

## Data Availability

The data that support the findings of this study are openly available in OSF at https://doi.org/10.17605/OSF.IO/KV3R9.

## References

[jora70114-bib-0001] Achenbach, T. M. , Ivanova, M. Y. , Rescorla, L. A. , Turner, L. V. , & Althoff, R. R. (2016). Internalizing/externalizing problems: Review and recommendations for clinical and research applications. Journal of the American Academy of Child and Adolescent Psychiatry, 55(8), 647–656. 10.1016/j.jaac.2016.05.012 27453078

[jora70114-bib-0086] Adler, N. E. , Epel, E. S. , Castellazzo, G. , & Ickovics, J. R. (2000). Relationship of subjective and objective social status with psychological and physiological functioning: Preliminary data in healthy, White women. Health Psychology, 19(6), 586–592. 10.1037/0278-6133.19.6.586 11129362

[jora70114-bib-0002] Aldao, A. , Nolen‐Hoeksema, S. , & Schweizer, S. (2010). Emotion‐regulation strategies across psychopathology: A meta‐analytic review. Clinical Psychology Review, 30(2), 217–237. 10.1016/j.cpr.2009.11.004 20015584

[jora70114-bib-0087] Aldao, A. , Sheppes, G. , & Gross, J. J. (2015). Emotion regulation flexibility. Cognitive Therapy and Research, 39(3), 263–278. 10.1007/s10608-014-9662-4

[jora70114-bib-0003] Arend, M. G. , & Schäfer, T. (2019). Statistical power in two‐level models: A tutorial based on Monte Carlo simulation. Psychological Methods, 24(1), 1. 30265048 10.1037/met0000195

[jora70114-bib-0004] Asparouhov, T. , Hamaker, E. L. , & Muthén, B. (2018). Dynamic structural equation models. Structural Equation Modeling: A Multidisciplinary Journal, 25(3), 359–388. 10.1080/10705511.2017.1406803

[jora70114-bib-0005] Asparouhov, T. , & Muthén, B. (2010). Bayesian analysis of latent variable models using Mplus.

[jora70114-bib-0006] Asparouhov, T. , & Muthén, B. (2019). Latent variable centering of predictors and mediators in multilevel and time‐series models. Structural Equation Modeling: A Multidisciplinary Journal, 26(1), 119–142.

[jora70114-bib-0007] Baer, R. A. , Smith, G. T. , Hopkins, J. , Krietemeyer, J. , & Toney, L. (2006). Using self‐report assessment methods to explore facets of mindfulness. Assessment, 13(1), 27–45. 16443717 10.1177/1073191105283504

[jora70114-bib-0008] Bai, S. , Elavsky, S. , Kishida, M. , Dvořáková, K. , & Greenberg, M. T. (2020). Effects of mindfulness training on daily stress response in college students: Ecological momentary assessment of a randomized controlled trial. Mindfulness, 11(6), 1–13. 10.1007/s12671-020-01358-x PMC7748211 33343764

[jora70114-bib-0009] Bitsko, R. H. , Holbrook, J. R. , Ghandour, R. M. , Blumberg, S. J. , Visser, S. N. , Perou, R. , & Walkup, J. T. (2018). Epidemiology and impact of health care provider–diagnosed anxiety and depression among US children. Journal of Developmental and Behavioral Pediatrics, 39(5), 395. 29688990 10.1097/DBP.0000000000000571 PMC6003874

[jora70114-bib-0010] Boden, M. T. , Irons, J. G. , Feldner, M. T. , Bujarski, S. , & Bonn‐Miller, M. O. (2015). An investigation of relations among quality of life and individual facets of emotional awareness and mindfulness. Mindfulness, 6(4), 700–707. 10.1007/s12671-014-0308-0

[jora70114-bib-0011] Brockman, R. , Ciarrochi, J. , Parker, P. , & Kashdan, T. (2017). Emotion regulation strategies in daily life: Mindfulness, cognitive reappraisal and emotion suppression. Cognitive Behaviour Therapy, 46(2), 91–113. 27684649 10.1080/16506073.2016.1218926

[jora70114-bib-0012] Brown, K. W. , Goodman, R. J. , & Inzlicht, M. (2013). Dispositional mindfulness and the attenuation of neural responses to emotional stimuli. Social Cognitive and Affective Neuroscience, 8(1), 93–99. 10.1093/scan/nss004 22253259 PMC3541486

[jora70114-bib-0013] Brown, K. W. , & Ryan, R. M. (2003). The benefits of being present: Mindfulness and its role in psychological well‐being. Journal of Personality and Social Psychology, 84(4), 822–848. 10.1037/0022-3514.84.4.822 12703651

[jora70114-bib-0014] Chavira, D. A. , Ponting, C. , & Ramos, G. (2022). The impact of COVID‐19 on child and adolescent mental health and treatment considerations. Behaviour Research and Therapy, 157, 104169. 35970084 10.1016/j.brat.2022.104169 PMC9339162

[jora70114-bib-0015] Chen, M. , & Cheung, R. Y. M. (2021). Testing interdependent self‐construal as a moderator between mindfulness, emotion regulation, and psychological health among emerging adults. International Journal of Environmental Research and Public Health, 18(2), 2. 10.3390/ijerph18020444 PMC7827106 33429953

[jora70114-bib-0016] Chiesa, A. , Anselmi, R. , & Serretti, A. (2014). Psychological mechanisms of mindfulness‐based interventions: What do we know? Holistic Nursing Practice, 28(2), 124–148. 24503749 10.1097/HNP.0000000000000017

[jora70114-bib-0017] Coffey, K. A. , Hartman, M. , & Fredrickson, B. L. (2010). Deconstructing mindfulness and constructing mental health: Understanding mindfulness and its mechanisms of action. Mindfulness, 1(4), 235–253. 10.1007/s12671-010-0033-2

[jora70114-bib-0018] Cohen, J. (2013). Statistical power analysis for the behavioral sciences. Routledge.

[jora70114-bib-0019] Coles, M. E. , Turk, C. L. , Jindra, L. , & Heimberg, R. G. (2004). The path from initial inquiry to initiation of treatment for social anxiety disorder in an anxiety disorders specialty clinic. Journal of Anxiety Disorders, 18(3), 371–383. 10.1016/S0887-6185(02)00259-1 15125983

[jora70114-bib-0020] Cortazar, N. , & Calvete, E. (2019). Dispositional mindfulness and its moderating role in the predictive association between stressors and psychological symptoms in adolescents. Mindfulness, 10(10), 2046–2059. 10.1007/s12671-019-01175-x

[jora70114-bib-0021] Creswell, J. D. , & Lindsay, E. K. (2014). How does mindfulness training affect health? A mindfulness stress buffering account. Current Directions in Psychological Science, 23(6), 401–407. 10.1177/0963721414547415

[jora70114-bib-0022] Creswell, J. D. , Way, B. M. , Eisenberger, N. I. , & Lieberman, M. D. (2007). Neural correlates of dispositional mindfulness during affect labeling. Psychosomatic Medicine, 69(6), 560–565. 17634566 10.1097/PSY.0b013e3180f6171f

[jora70114-bib-0023] Curran, P. J. (2003). Have multilevel models been structural equation models all along? Multivariate Behavioral Research, 38(4), 529–569. 10.1207/s15327906mbr3804_5 26777445

[jora70114-bib-0024] Curtiss, J. , Klemanski, D. H. , Andrews, L. , Ito, M. , & Hofmann, S. G. (2017). The conditional process model of mindfulness and emotion regulation: An empirical test. Journal of Affective Disorders, 212, 93–100. 10.1016/j.jad.2017.01.027 28157552 PMC5340204

[jora70114-bib-0025] Deng, X. , Gao, Q. , Zhang, L. , & Li, Y. (2020). Neural underpinnings of the role of trait mindfulness in emotion regulation in adolescents. Mindfulness, 11(5), 1120–1130. 10.1007/s12671-019-01276-7

[jora70114-bib-0026] Deng, X. , Yang, M. , & An, S. (2021). Differences in frontal EEG asymmetry during emotion regulation between high and low mindfulness adolescents. Biological Psychology, 158, 107990. 10.1016/j.biopsycho.2020.107990 33279594

[jora70114-bib-0027] Didonna, F. , & Gonzalez, Y. R. (2009). Mindfulness and feelings of emptiness. In Clinical handbook of mindfulness (pp. 125–151). Springer.

[jora70114-bib-0028] Enders, C. K. (2023). Missing data: An update on the state of the art. Psychological Methods, 30(2), 322–339. 10.1037/met0000563. 36931827

[jora70114-bib-0029] Enkema, M. C. , McClain, L. , Bird, E. R. , Halvorson, M. A. , & Larimer, M. E. (2020). Associations between mindfulness and mental health outcomes: A systematic review of ecological momentary assessment research. Mindfulness, 11(11), 2455–2469. 10.1007/s12671-020-01442-2 35694042 PMC9187214

[jora70114-bib-0030] Foa, E. B. , Johnson, K. M. , Feeny, N. C. , & Treadwell, K. R. H. (2001). The child PTSD symptom scale: A preliminary examination of its psychometric properties. Journal of Clinical Child & Adolescent Psychology, 30(3), 376–384. 10.1207/S15374424JCCP3003_9 11501254

[jora70114-bib-0031] Fogarty, F. A. , Lu, L. M. , Sollers, J. J. , Krivoschekov, S. G. , Booth, R. J. , & Consedine, N. S. (2015). Why it pays to be mindful: Trait mindfulness predicts physiological recovery from emotional stress and greater differentiation among negative emotions. Mindfulness, 6(2), 175–185.

[jora70114-bib-0032] Forner, C. (2019). What mindfulness can learn about dissociation and what dissociation can learn from mindfulness. Journal of Trauma & Dissociation, 20(1), 1–15. 30095378 10.1080/15299732.2018.1502568

[jora70114-bib-0033] Garland, E. L. , & Fredrickson, B. L. (2013). Mindfulness broadens awareness and builds meaning at the attention‐emotion interface . Mindfulness, acceptance, and positive psychology: The seven foundations of well‐being, 30–67.

[jora70114-bib-0034] Gelman, A. , Carlin, J. B. , Stern, H. S. , & Rubin, D. B. (1995). Bayesian data analysis. Chapman and Hall/CRC.

[jora70114-bib-0036] Gratz, K. L. , & Roemer, L. (2004). Multidimensional assessment of emotion regulation and dysregulation: Development, factor structure, and initial validation of the difficulties in emotion regulation scale. Journal of Psychopathology and Behavioral Assessment, 26, 41–54. 10.1023/B:JOBA.0000007455.08539.94

[jora70114-bib-0037] Greco, L. A. , Baer, R. A. , & Smith, G. T. (2011). Assessing mindfulness in children and adolescents: Development and validation of the child and adolescent mindfulness measure (CAMM). Psychological Assessment, 23(3), 606–614. 10.1037/a0022819 21480722

[jora70114-bib-0088] Gross, J. J. (2014). Emotion regulation: Conceptual and empirical foundations. Handbook of Emotion Regulation, 2(1), 3–20.

[jora70114-bib-0038] Gross, J. J. (2015). The extended process model of emotion regulation: Elaborations, applications, and future directions. Psychological Inquiry, 26(1), 130–137. 10.1080/1047840X.2015.989751

[jora70114-bib-0039] Guendelman, S. , Medeiros, S. , & Rampes, H. (2017). Mindfulness and emotion regulation: Insights from neurobiological, psychological, and clinical studies. Frontiers in Psychology, 8, 220. 28321194 10.3389/fpsyg.2017.00220 PMC5337506

[jora70114-bib-0040] Hall, S. P. (2020). Being mindful about mindfulness: Exploring the dark side. International Journal of Coercion Abuse & Manipulation, 1(1), 17–28.

[jora70114-bib-0041] Hambour, V. K. , Zimmer‐Gembeck, M. J. , Clear, S. , Rowe, S. , & Avdagic, E. (2018). Emotion regulation and mindfulness in adolescents: Conceptual and empirical connection and associations with social anxiety symptoms. Personality and Individual Differences, 134, 7–12. 10.1016/j.paid.2018.05.037

[jora70114-bib-0042] Heppner, W. L. , Kernis, M. H. , Lakey, C. E. , Campbell, W. K. , Goldman, B. M. , Davis, P. J. , & Cascio, E. V. (2008). Mindfulness as a means of reducing aggressive behavior: Dispositional and situational evidence. Aggressive Behavior, 34(5), 486–496. 18464229 10.1002/ab.20258

[jora70114-bib-0043] Heron, K. E. , Everhart, R. S. , McHale, S. M. , & Smyth, J. M. (2017). Using mobile‐technology‐based ecological momentary assessment (EMA) methods with youth: A systematic review and recommendations. Journal of Pediatric Psychology, 42(10), 1087–1107. 10.1093/jpepsy/jsx078 28475765

[jora70114-bib-0044] Jankowski, T. , & Holas, P. (2014). Metacognitive model of mindfulness. Consciousness and Cognition, 28, 64–80. 10.1016/j.concog.2014.06.005 25038535

[jora70114-bib-0045] Kabat‐Zinn, J. (1994). Wherever you go, there you are: Mindfulness meditation in everyday life. Hyperion.

[jora70114-bib-0046] Keng, S.‐L. , Smoski, M. J. , Robins, C. J. , Ekblad, A. G. , & Brantley, J. G. (2012). Mechanisms of change in mindfulness‐based stress reduction: Self‐compassion and mindfulness as mediators of intervention outcomes. Journal of Cognitive Psychotherapy, 26(3), 270–280. 10.1891/0889-8391.26.3.270

[jora70114-bib-0047] Keng, S.‐L. , & Tong, E. M. W. (2016). Riding the tide of emotions with mindfulness: Mindfulness, affect dynamics, and the mediating role of coping. Emotion, 16(5), 706. 10.1037/emo0000165 27064290

[jora70114-bib-0048] Kreft, I. G. G. , & De Leeuw, J. (1998). Introducing multilevel modeling. SAGE Publications, Ltd. 10.4135/9781849209366

[jora70114-bib-0049] Lavender, J. M. , Tull, M. T. , DiLillo, D. , Messman‐Moore, T. , & Gratz, K. L. (2017). Development and validation of a state‐based measure of emotion dysregulation: The state difficulties in emotion regulation scale (S‐DERS). Assessment, 24(2), 197–209. 10.1177/1073191115601218 26297011 PMC5243864

[jora70114-bib-0050] Lewis, C. , Roberts, N. P. , Gibson, S. , & Bisson, J. I. (2020). Dropout from psychological therapies for post‐traumatic stress disorder (PTSD) in adults: Systematic review and meta‐analysis. European Journal of Psychotraumatology, 11(1), 1709709. 10.1080/20008198.2019.1709709 32284816 PMC7144189

[jora70114-bib-0051] Lindsay, E. K. , & Creswell, J. D. (2017). Mechanisms of mindfulness training: Monitor and acceptance theory (MAT). Clinical Psychology Review, 51, 48–59. 27835764 10.1016/j.cpr.2016.10.011 PMC5195874

[jora70114-bib-0052] Lindsay, E. K. , & Creswell, J. D. (2018). Mindfulness, acceptance, and emotion regulation: Perspectives from monitor and acceptance theory (MAT). Current Opinion in Psychology, 28, 120–125. 30639835 10.1016/j.copsyc.2018.12.004 PMC6565510

[jora70114-bib-0053] Liu, X. , Shi, S. , Wen, X. , Chen, J. , & Xu, W. (2022). Mindfulness and posttraumatic response patterns among adolescents following the tornado. Children and Youth Services Review, 134, 106375. 10.1016/j.childyouth.2022.106375

[jora70114-bib-0090] Lucas‐, R. G. , Thompson , Miller, R. L. , Seiter, N. S. , Prince, M. A. , Crain, T. L. , & Shomaker, L. B. (2021). Within‐Person variations in mindfulness mediate effects of daily stressors on psychological distress in adolescence. Psychology & Health, 37(9), 1057–1075. 10.1080/08870446.2021.1929982 34139904 PMC10569682

[jora70114-bib-0054] Ma, Y. , & Fang, S. (2019). Adolescents' mindfulness and psychological distress: The mediating role of emotion regulation. Frontiers in Psychology, 10, 430770. 10.3389/fpsyg.2019.01358 PMC6567674 31231292

[jora70114-bib-0055] McNeish, D. , & Hamaker, E. L. (2020). A primer on two‐level dynamic structural equation models for intensive longitudinal data in Mplus. Psychological Methods, 25(5), 610. 31855015 10.1037/met0000250

[jora70114-bib-0056] Miller, R. L. , Shomaker, L. B. , Prince, M. A. , Haddock, S. , Rzonca, A. , Krause, J. T. , Zimmerman, T. , Lavender, J. M. , Sibinga, E. , & Lucas‐Thompson, R. G. (2024). Momentary effects of life stressors on mindfulness and emotion regulation difficulties among adolescents exposed to chronic stressors. Stress and Health, 40(5), e3414. 10.1002/smi.3414 38685855 PMC13426062

[jora70114-bib-0057] Moore, R. C. , Depp, C. A. , Wetherell, J. L. , & Lenze, E. J. (2016). Ecological momentary assessment versus standard assessment instruments for measuring mindfulness, depressed mood, and anxiety among older adults. Journal of Psychiatric Research, 75, 116–123. 10.1016/j.jpsychires.2016.01.011 26851494 PMC4769895

[jora70114-bib-0058] Morrison, A. B. , & Jha, A. P. (2015). Mindfulness, attention, and working memory. In Handbook of mindfulness and self‐regulation (pp. 33–45). Springer Science + Business Media. 10.1007/978-1-4939-2263-5_4

[jora70114-bib-0059] Morrison, W. , Guerdan, L. , Kanugo, J. , Trull, T. , & Shang, Y. (2018). Tigeraware: An innovative mobile survey and sensor data collection and analytics system. 2018 IEEE Third International Conference on Data Science in Cyberspace (DSC), 115–122.

[jora70114-bib-0060] Muthén, B. , Asparouhov, T. , & Shiffman, S. (2025). Dynamic structural equation modeling with floor effects. Psychological Methods. Advance online publication. 10.1037/met0000720 39760732

[jora70114-bib-0061] Muthén, L. K. , & Muthén, B. O. (2017). Mplus User's Guide (Eight ed.). Muthén & Muthén.

[jora70114-bib-0062] Pepping, C. A. , Duvenage, M. , Cronin, T. J. , & Lyons, A. (2016). Adolescent mindfulness and psychopathology: The role of emotion regulation. Personality and Individual Differences, 99, 302–307. 10.1016/j.paid.2016.04.089

[jora70114-bib-0063] Preacher, K. J. , Zhang, Z. , & Zyphur, M. J. (2016). Multilevel structural equation models for assessing moderation within and across levels of analysis. Psychological Methods, 21(2), 189. 26651982 10.1037/met0000052

[jora70114-bib-0064] Roemer, L. , Williston, S. K. , & Rollins, L. G. (2015). Mindfulness and emotion regulation. Current Opinion in Psychology, 3, 52–57. 10.1016/j.copsyc.2015.02.006

[jora70114-bib-0065] Röll, J. , Koglin, U. , & Petermann, F. (2012). Emotion regulation and childhood aggression: Longitudinal associations. Child Psychiatry & Human Development, 43(6), 909–923. 10.1007/s10578-012-0303-4 22528031

[jora70114-bib-0066] Royuela‐Colomer, E. , Fernández‐González, L. , & Orue, I. (2021). Longitudinal associations between internalizing symptoms, dispositional mindfulness, rumination and impulsivity in adolescents. Journal of Youth and Adolescence, 50(10), 2067–2078. 10.1007/s10964-021-01476-2 34244923 PMC8416885

[jora70114-bib-0067] Schmitz, J. C. S. , Prenoveau, J. M. , Papadakis, A. A. , Johnson, A. J. , Lating, J. M. , Mendelson, T. , & Dariotis, J. K. (2021). Mindfulness and posttraumatic stress disorder symptom severity in urban African‐American high school students. Psychiatric Quarterly, 92(1), 85–99. 10.1007/s11126-020-09774-x 32458341

[jora70114-bib-0068] Schwarz, N. (2007). Retrospective and concurrent self‐reports: The rationale for real‐time data capture. In The science of real‐time data capture: Self‐reports in health research (pp. 11–26). Oxford University Press.

[jora70114-bib-0069] Seah, R. , Dwyer, K. , & Berle, D. (2023). Was it me? The role of attributions and shame in posttraumatic stress disorder (PTSD): A systematic review. Trends in Psychology, 33, 783–804. 10.1007/s43076-023-00315-6

[jora70114-bib-0070] Sheppes, G. , Suri, G. , & Gross, J. J. (2015). Emotion regulation and psychopathology. Annual Review of Clinical Psychology, 11, 379–405. 10.1146/annurev-clinpsy-032814-112739 25581242

[jora70114-bib-0071] Shiffman, S. , Stone, A. A. , & Hufford, M. R. (2008). Ecological momentary assessment. Annual Review of Clinical Psychology, 4, 1–32. 10.1146/annurev.clinpsy.3.022806.091415 18509902

[jora70114-bib-0072] Shin, K. E. , Newman, M. G. , & Jacobson, N. C. (2022). Emotion network density is a potential clinical marker for anxiety and depression: Comparison of ecological momentary assessment and daily diary. British Journal of Clinical Psychology, 61, 31–50. 10.1111/bjc.12295 33963538 PMC8572316

[jora70114-bib-0073] Siebelink, N. M. , Asherson, P. , Antonova, E. , Bögels, S. M. , Speckens, A. E. , Buitelaar, J. K. , & Greven, C. U. (2019). Genetic and environmental aetiologies of associations between dispositional mindfulness and ADHD traits: A population‐based twin study. European Child & Adolescent Psychiatry, 28(9), 1241–1251. 10.1007/s00787-019-01279-8 30758734 PMC6751144

[jora70114-bib-0074] Steinberg, L. (2014). Age of opportunity: Lessons from the new science of adolescence. Houghton Mifflin Harcourt.

[jora70114-bib-0075] Sutton, E. , Schonert‐Reichl, K. A. , Wu, A. D. , & Lawlor, M. S. (2018). Evaluating the reliability and validity of the self‐compassion scale short form adapted for children ages 8–12. Child Indicators Research, 11(4), 1217–1236. 10.1007/s12187-017-9470-y

[jora70114-bib-0076] Teper, R. , Segal, Z. V. , & Inzlicht, M. (2013). Inside the mindful mind: How mindfulness enhances emotion regulation through improvements in executive control. Current Directions in Psychological Science, 22(6), 449–454.

[jora70114-bib-0077] Vujanovic, A. A. , Bonn‐Miller, M. O. , Bernstein, A. , McKee, L. G. , & Zvolensky, M. J. (2010). Incremental validity of mindfulness skills in relation to emotional dysregulation among a Young adult community sample. Cognitive Behaviour Therapy, 39(3), 203–213. 10.1080/16506070903441630 20182933 PMC2889232

[jora70114-bib-0078] Warren, M. T. , Wray‐Lake, L. , & Shubert, J. (2020). Developmental changes in mindful awareness during adolescence. International Journal of Behavioral Development, 44(1), 31–40. 10.1177/0165025419885023

[jora70114-bib-0079] Wen, C. K. F. , Schneider, S. , Stone, A. A. , & Spruijt‐Metz, D. (2017). Compliance with mobile ecological momentary assessment protocols in children and adolescents: A systematic review and meta‐analysis. Journal of Medical Internet Research, 19(4), e132. 10.2196/jmir.6641 28446418 PMC5425774

[jora70114-bib-0080] Whitney, D. G. , & Peterson, M. D. (2019). US national and state‐level prevalence of mental health disorders and disparities of mental health care use in children. JAMA Pediatrics, 173(4), 389–391. 30742204 10.1001/jamapediatrics.2018.5399 PMC6450272

[jora70114-bib-0081] Wickham, H. , & Sievert, C. (2009). ggplot2: Elegant graphics for data analysis (Vol. 10). springer New York.

[jora70114-bib-0082] Wright, I. , Mughal, F. , Bowers, G. , & Meiser‐Stedman, R. (2021). Dropout from randomised controlled trials of psychological treatments for depression in children and youth: A systematic review and meta‐analyses. Journal of Affective Disorders, 281, 880–890. 33248810 10.1016/j.jad.2020.11.039

[jora70114-bib-0083] Young, K. S. , Sandman, C. F. , & Craske, M. G. (2019). Positive and negative emotion regulation in adolescence: Links to anxiety and depression. Brain Sciences, 9(4), 76. 10.3390/brainsci9040076 30934877 PMC6523365

[jora70114-bib-0084] Zhang, D. , Lee, E. K. P. , Mak, E. C. W. , Ho, C. Y. , & Wong, S. Y. S. (2021). Mindfulness‐based interventions: An overall review. British Medical Bulletin, 138(1), 41–57. 10.1093/bmb/ldab005 33884400 PMC8083197

[jora70114-bib-0085] Zhang, W. , Ouyang, Y. , Tang, F. , Chen, J. , & Li, H. (2019). Breath‐focused mindfulness alters early and late components during emotion regulation. Brain and Cognition, 135, 103585. 10.1016/j.bandc.2019.103585 31374347

