# Supplementary material for: Exploring within and between associations of momentary mindfulness and emotion regulation and the moderating effects of mental health among adolescents
Source: J Res Adolesc. 2026 Jan 1;36(1):e70114. doi: 10.1111/jora.70114 (PMC12757435; doi:10.1111/jora.70114)
Supplement: Supplementary file 1 — Table S1. Descriptive statistics for key study variables. [file JORA-36-0-s001.docx]

**Supplemental Table 1**

*Descriptive statistics for key study variables*

|  | *M^a^* | *SD* | Range | ICCs^b^ |
| --- | --- | --- | --- | --- |
| Mindful Attention | 5.52 | 1.16 | 2.60-7 | .58 |
| Mindful Non-judgment | 5.08 | 1.61 | 1-7 | .71 |
| Difficulties with Emotion Regulation | 2.31 | 1.24 | 1-6.50 | .68 |
| PTSD symptoms | 20.52 | 13.30 | 0-50 | -- |
| Internalizing Symptoms | 5.40 | 3.57 | 0-12 | -- |
| Externalizing Symptoms | 3.90 | 2.35 | 0-12 | -- |
| Attention Problems | 6.36 | 2.35 | 1-12 | -- |

*Note.* ^a^Accounts for clustering within person. ^b^The grouping variable within analyses for intraclass correlations (ICCS) was participant.
